# Predictors of HbA1c among Adipocytokine Biomarkers in African-American Men with Varied Glucose Tolerance

**DOI:** 10.3390/biomedicines8110520

**Published:** 2020-11-20

**Authors:** Elena Barengolts, Arfana Akbar, Brian T. Layden, Yuval Eisenberg, Medha Priyadarshini, Jeffrey A. Borgia, Cristina L. Fhied, Michael Salim, Lara R. Dugas

**Affiliations:** 1 Division of Endocrinology, Diabetes and Metabolism, University of Illinois at Chicago School of Medicine, Chicago, IL 60612, USA; blayde1@uic.edu (B.T.L.); eisenbe1@uic.edu (Y.E.); mpriya2@uic.edu (M.P.); 2 Section of Endocrinology, Jesse Brown Veterans Affairs Medical Center, Chicago, IL 60612, USA; arfana.akbar@va.gov; 3 Department of Cell & Molecular Medicine, Rush University Medical Center, Chicago, IL 60612, USA; Jeffrey_A_Borgia@rush.edu; 4 Department of Pathology, Rush University Medical Center, Chicago, IL 60612, USA; cristina_l_fhied@rush.edu; 5 Department of Internal Medicine, Mount Sinai Hospital, Chicago, IL 60608, USA; michael.salim@sinai.org; 6 Public Health Sciences, Parkinson School of Health Sciences and Public Health, Loyola University Chicago, Maywood, IL 60153, USA; ldugas@luc.edu

**Keywords:** type 2 diabetes, cytokines, adipokines, adipsin, TNF-α, multiplex assay

## Abstract

This study explored adipocytokine associations with acute and chronic hyperglycemia in African-American men (AAM). Fourteen adipocytokines were measured from men with normal glucose tolerance (NGT) or type 2 diabetes (T2D, drug-naïve MF(−) or using metformin MF(+)). Acute and chronic hyperglycemia were evaluated by 120 min oral glucose tolerance test (OGTT) and glycohemoglobin A1c (HbA1c). AAM with T2D (*n* = 21) compared to NGT (*n* = 20) were older, had higher BMI and slightly higher glucose and insulin. In the fasted state, TNF-α, IL-6, PAI-1, IL-13, adiponectin, adipsin, and lipocalin were lower in T2D vs. NGT. At 120 min post-glucose load, TNF-α, IL-6, IL-13, IL-8, PAI-1, adiponectin, adipsin, lipocalin, and resistin were lower in T2D vs. NGT. There were no statistical differences for GM-CSF, IL-7, IL-10, IP-10, and MCP-1. Regression analysis showed that fasting IL-8, TNF-α, adiponectin, lipocalin, resistin, adipsin, and PAI-1 were associated with HbA1c. After adjusting for age, BMI, glucose tolerance, and metformin use, only adipsin remained significantly associated with HbA1c (*p* = 0.021). The model including adipsin, TNF-α, age, BMI, and group designation (i.e., NGT, MF(−), MF(+)) explained 86% of HbA1c variability. The data suggested that adipsin could be associated with HbA1c in AAM with varied glucose tolerance.

## 1. Introduction

Type 2 diabetes (T2D) is a common and emergent problem in the USA and worldwide. More than 30 million Americans have diabetes (about 1 in 10), and 90% to 95% of them have T2D [1]. In the last 20 years, the number of adults diagnosed with T2D has more than doubled as the American population has aged and become more overweight or obese [1]. Globally, the number of people with T2D has risen from 108 million in 1980 to 422 million in 2014 according to the World Health Organization (WHO) [2]. In the United States and worldwide, T2D is estimated to be the seventh leading cause of death [1,2]. Globally in 2016, an estimated 1.6 million deaths were attributed to diabetes, with death commonly occurring before the age of 70 years [2].

T2D management remains a challenge despite of recent significant advances [3,4,5]. Part of this challenge includes the multifactorial metabolic confluence contributing to glucose homeostasis [6]. Among these factors, inflammation-related proteins cytokines [7,8,9,10,11,12] and adipose-tissue-secreted proteins adipokines [13,14,15,16] appear to play important roles. Notably, numerous studies have explored the hypothesis that inflammation and adiposity may contribute to T2D by investigating cell signaling, animal models, and gene knockout models [7,17,18]. The results of these studies support the concept that T2D involves adipocytokines as mediators of low-grade subclinical inflammatory processes contributing to the pathogenesis of T2D at all stages, i.e., from development to progression, complications, and mortality [7,8,9,19,20,21,22,23]. To date, however, clinical trials have failed to translate these discoveries into T2D treatment [24,25,26]. A recently developed new methodology enables the search for novel adipocytokines that could be important for advancing T2D management to be expanded. 

Adipokines are secreted by white adipose tissue and have been found to be active contributors to glucose homeostasis [13,14,15,16]. Among these adipokines, adipsin, resistin, and lipocalin-2 are implicated as important mediators of inflammation [11,13,27]. There are also data suggesting that the degree of adipose tissue inflammation, not obesity per se, is a precondition for the development of insulin resistance in T2D [28,29]. Plasminogen activator inhibitor 1 (PAI-1) can likewise be included into the adipokine family of proteins since it is secreted in substantial quantities by adipose tissue [14,27,30]. PAI-1, a well-known regulator of fibrinolytic system, is also implicated in inflammation-related T2D complications such as cardiovascular disease [11,20,31]. 

Cytokines are small proteins secreted by a variety of cells, including stromal and other cells (e.g., lymphocytes, macrophages) in adipose tissue that can act in an autocrine, paracrine, and hormone-like fashion to promote or reduce inflammatory processes [7,8,9,10,11]. Cytokines that promote inflammation include interleukins (IL) IL-7 and IL-8, granulocyte–macrophage colony-stimulating factor (GM-CSF), interferon-inducible protein-10 (IP-10), monocyte chemoattractant protein (MCP)-1, and tumor necrosis factor (TNF)-α, among others [7,8,9,27]. Cytokines that reduce inflammation include IL-10 and IL-13 [32,33], while IL-6 has been implicated as a pro- and anti-inflammatory mediator [34].

**Figure 1:**
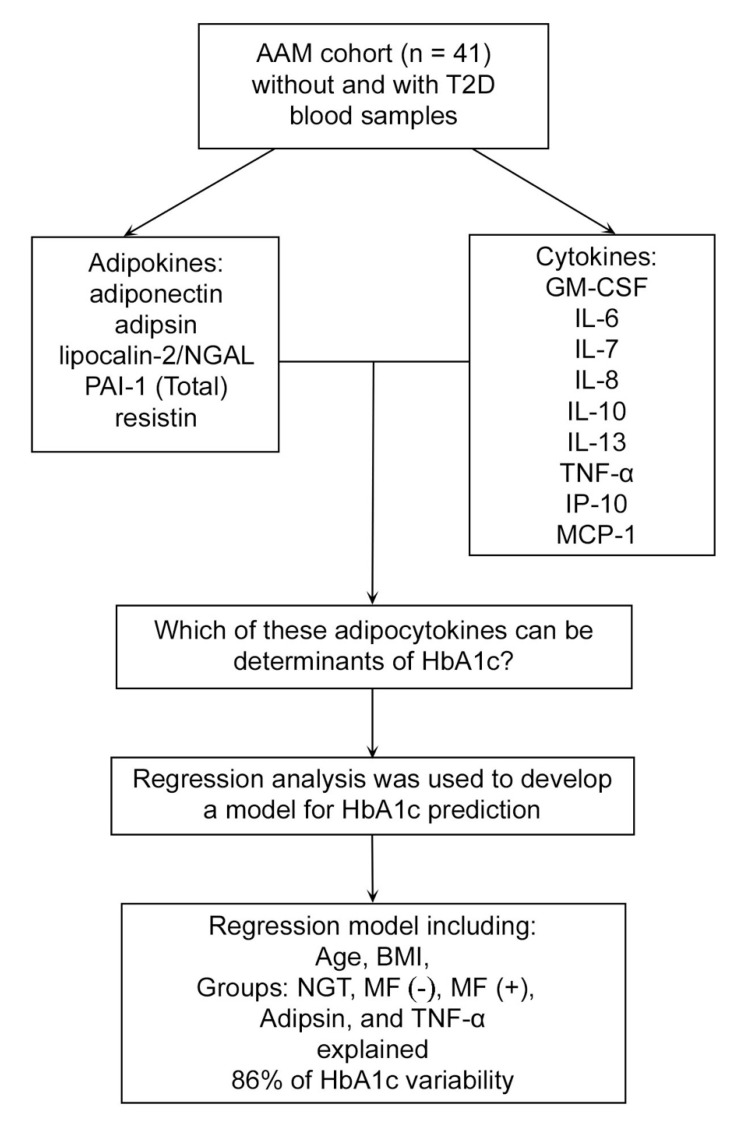
Study design and main results. Abbreviations: AAM, African-American men; BMI, body mass index; MF, metformin; NGT, normal glucose tolerance; T2D, type 2 diabetes.

Recent advances in high-sensitivity bead-based multiplex assay technologies [11,35,36,37] have resulted in the simultaneous measurement of adipocytokines from a single blood sample with high accuracy and reproducibility. These proteomic multiplexed immunoassays have gained widespread applications for detecting key biomarkers to provide mechanistic insight into various conditions, including T2D and its complications [11,36,37,38]. The multiplex methodology can improve individualized medical decision-making based on biomarker profiles, in addition to specific clinical risk factors, as suggested by data for cardiovascular disease [11,36,37,38]. A gap in knowledge, however, remains in understanding whether individual adipocytokines are associated with acute or chronic hyperglycemia in human studies. We previously observed varied responses of 35 biomarkers to acute hyperglycemia in men with prediabetes [39], while other studies also observed inconsistent associations of selected adipocytokines with acute (first phase) insulin response [40] and glycohemoglobin A1c (HbA1c) [41] in diabetic individuals. Therefore, the primary objective of this pilot study was to explore whether the adipocytokine responses (measured by multiplex assay) to acute hyperglycemia differed by T2D status during an oral glucose tolerance test (OGTT). Secondly, we explored whether fasting adipocytokines were associated with chronic hyperglycemia measured by HbA1c (Figure 1).

## 2. Experimental Section

### 2.1. Design and Participants 

The current analysis was performed as a subanalysis in the cross-sectional study of glucose tolerance and vitamin D insufficiency in African-American men (AAM), investigating glucose metabolism and selected biomarker interactions in AAM [42,43]. The original study was conducted at an urban Veteran Administration Medical Center (VAMC). The study was approved by the Jesse Brown VA Medical Center (JBVAMC) Institutional Review Board (the project identification code: 2012-0272, date 06 Feb, 2013). The intended subject population (*n* = 100) included those with T2D (*n* = 50) and those without T2D (NGT, normal glucose tolerance participants, *n* = 50) [42,43]. The inclusion criteria were HbA1c < 5.7% for NGT or 6.5–7.4% for T2D, age 35–70 years, body mass index (BMI) 22–39.9 kg/m^2^, and 25OH-vitamin D (25OHD) < 30 ng/mL. Diabetic participants were allowed to participate so long as they were non-medically treated (lifestyle modification) or using metformin alone. Exclusion criteria were chronic kidney disease (stages 3b, 4, and 5), chronic glucocorticoid use (3 months or longer), taking non-metformin antihyperglycemic medications, significant T2D complications, and health conditions requiring recent (within 6 months) hospitalization. The participants came for a single study visit after an overnight fast. The research staff reviewed past medical history and medication use. The OGTT using 75 g glucose load was performed and blood was collected for biomarker analysis as previously described [42,43]. For this pilot study, blood samples for analysis of adipocytokines were chosen at random based on the availability of samples at 0 and 120 min of OGTT. The final sample included 41 participants, *n* = 20 with normal glucose tolerance, *n* = 21 with T2D. The recruitment dates were from December 01, 2013, to April 15, 2016 (Figure 1).

### 2.2. Biometrics and Glycemic Measures 

Biometrics including weight (kg), height (m), and calculated BMI (kg/m^2^) were performed using study-specific standardized techniques [42,43]. The age-adjusted Charlson index of chronic disease was calculated as previously described [42,43]. Glycemic control was assessed by HbA1c, as well as fasting and 2 h (120 min) post-glucose load glucose and insulin [42,43,44]. Blood glucose, insulin, and HbA1c were measured in the Clinical Laboratory Improvement Amendments (CLIA)-approved clinical laboratory by applying laboratory standards of care as previously described [42,43,44]. 

### 2.3. Adipocytokine Biomarker Analysis

Blood for biomarkers was collected during OGTT and serum was stored at –80 °C until assayed. Adipocytokine biomarkers were measured at the Rush Biomarker Development Core Research Laboratory by applying laboratory standards of care as previously described [39,45,46]. A total of 14 serum adipocytokine biomarkers were explored in the Luminex immunobead platform using commercially available kits that were implemented according to the manufacturer’s recommended protocols. All primary data points were collected on a Luminex FLEXMAP 3D^®^ system (Luminex Corp., Austin, TX, USA) with concentrations calculated based on 7-point standard curves using a five-parametric fit algorithm in xPONENT^®^ v4.0.3 (Luminex Corp., Austin, TX, USA). All data met minimum quality control thresholds defined by the kit manufacturer, with percent coefficient of variation (%CV) values ≤ 10, as previously defined [45,46].

The cytokines including GM-CSF, IL-6, IL-7, IL-8, IL-10, IL-13, TNF-α, IP-10, and MCP-1 were analyzed using the MILLIPLEX MAP Human High-Sensitivity T Cell Panel—Immunology Multiplex Assay (catalog number HCYTOMAG-60K-01, Millipore Corporation, Billerica, MA, USA). The adipokines, including adiponectin, adipsin, lipocalin-2/NGAL, PAI-1 (Total), and resistin, were analyzed using MILLIPLEX MAP Human Adipokine Magnetic Bead Panel 1—Endocrine Multiplex Assay (catalog number HADK1MAG-61K-05, Millipore Corporation, Billerica, MA, USA). The multiplex bead technology has been confirmed to be highly reproducible and appropriately correlated with values obtained with classical enzyme-linked immunosorbent assays [47,48,49,50].

### 2.4. Statistical Analysis 

The current analysis explored the cross-sectional associations between adipocytokines at baseline (fasting state, 0 min OGTT) and following the 2 h post-glucose OGTT (i.e., acute hyperglycemia) in research participants grouped by chronic glycemic control (i.e., HbA1c). The groups were normal glucose tolerance (NGT) and T2D, either drug naïve for metformin (MF(−)) or taking metformin (MF(+)). It was expected that the patients prescribed metformin could differ from the patients not taking metformin. To explore this possibility, the comparison of NGT participants with MF(+) as well as MF(−) participants was performed even though the MF(−) group was very small (*n* = 5). Data were described as the mean ± standard deviation (SD) or standard error (SE) and log transformation was applied as appropriate for non-normal data distribution. To explore the independent associations between chronic glycemic control and adipocytokines we used linear regression analysis, adjusting for age and BMI. We then used backward stepwise multiple linear regression analysis to explore the relationship between chronic glycemic control (HbA1c) and fasting adipocytokines. Variables were excluded from the final model if the *p*-value > 0.1000. Post-estimation collinearity was probed using the variance inflation factor (VIF) for collinearity and 1/VIF for multicollinearity. All analyses were adjusted for age and BMI. A *p*-value of *p* < 0.05 was considered statistically significant. All analyses were performed in STATA (v.14, College Station, TX, USA).

## 3. Results

### 3.1. Study Population Characteristics and Glycemic Indices

The final sample included 41 participants, 20 with NGT and 21 with T2D. Sixteen of the T2D participants were taking metformin, while 5 were metformin-naïve. Men with T2D were significantly older, weighed more, and had higher BMI and HbA1c than participants with NGT (*p* < 0.01 for all) (Table 1). Fasting and 120 min post-glucose load glucose and insulin were higher in T2D than NGT, however the differences were not significant after adjusting for age and BMI. Overall, participants with T2D suffered a higher burden of disease as assessed by the Charlson index (*p* < 0.05) but were prescribed a similar number of medications. The results differed slightly when T2D participants were separated into taking metformin MF(+) or metformin-naïve MF(−). Specifically, MF(+) participants carried a significantly higher burden of disease (Charlson index: *p* < 0.01) while MF(−) group tended to be prescribed less medications (*p* = 0.07). Because of the age and adiposity differences, we adjusted all subsequent analyses for age and BMI. 

### 3.2. Adipocytokines and Diabetes Status 

To explore the effect of diabetes status on adipocytokines in both the fasted and 120 min post-glucose load states we used linear regression analysis, adjusting for age and BMI (Table 2). In the fasted state, i.e., before the glucose load (0 min OGTT), cytokines TNF-α and IL-6 (*p* < 0.01 for both) as well as IL-13 (*p* < 0.05) were lower in T2D compared to NGT. These differences remained among MF(+), while among MF(−), the cytokines TNF-α (*p* < 0.01) and IL-6 (*p* < 0.05) were significantly different. In addition, IL-8 was lower in MF(+) (*p* < 0.01) and MF(−) (*p* < 0.05) compared to NGT group. At 120 min post-glucose load, TNF-α, IL-6, IL-13, and IL-8 were lower in T2D compared to NGT (*p* < 0.01 for all). In the MF(+) group, TNF-α, IL-8, IL-13 (*p* < 0.01 for all), and IL-6 (*p* < 0.05) were lower compared to NGT group. In the MF(−) group, some of these cytokines were different, namely TNF-α (*p* < 0.01), as well as IL-6 and IL-8 (*p* < 0.05 for both). The other cytokines were not different between the groups (Table 2). 

Among the adipokines (Table 3) in fasting state, PAI-1 (*p* < 0.01), as well as adiponectin, adipsin, and lipocalin (*p* < 0.05), were lower in T2D compared to NGT and remained significantly lower at 120 min post-glucose load (*p* < 0.01 for PAI-1, adiponectin, adipsin, and *p* < 0.05 for lipocalin). In addition, resistin was decreased post-glucose load (*p* < 0.05) in T2D vs. NGT. These differences remained significant for MF(+) participants in both the fasted and 120 min post-glucose load states, but not for lipocalin and resistin at 120 min post-glucose load. Lastly, for MF(−) group in fasted state, PAI-1 and lipocalin were lower (*p* < 0.05), while post-glucose PAI-1, adiponectin, adipsin (*p* < 0.01 for all), and resistin (*p* < 0.05) were also lower compared to NGT. There were no statistical differences for the other adipokines (Table 3).

### 3.3. Adipocytokine Predictors of HbA1c 

To explore the independent associations between each of the 14 adipocytokines and chronic glycemic control in the fasted state, we used linear regression analysis, adjusting for age and BMI (Table 4). All variables were log-transformed because they were not normally distributed. The log values of the cytokines IL-8 (*p* = 0.032) and TNF-α (*p* < 0.001), as well as the adipokines adiponectin (*p* = 0.003), lipocalin (*p* = 0.005), resistin (*p* = 0.003), adipsin (*p* < 0.001), and PAI-1 (*p* < 0.001), were significantly associated with HbA1c after adjusting for age and BMI. 

**Figure 2:**
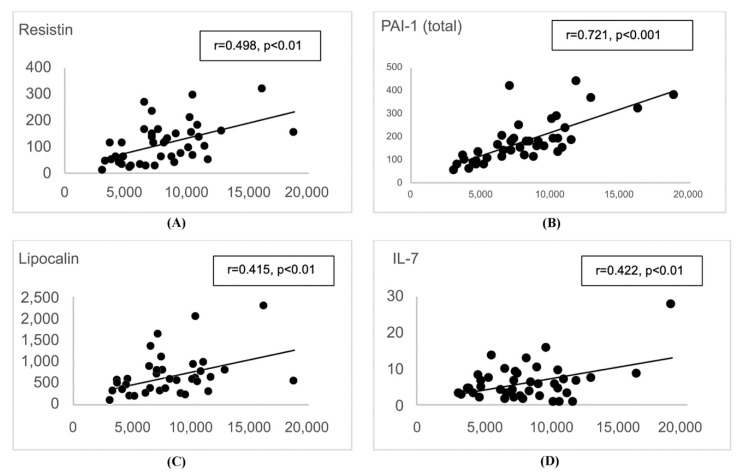
Results of Pearson correlation analysis between adipsin (*x* -axis) and adipocytokines (*y*-axis) for all participants (*n* = 41): (**A**) resistin; (**B**) PAI-1 (total); (**C**) lipocalin; (**D**) IL-7. Scatter plots for Pearson correlation coefficients (r) with *p* < 0.01.

Next, backwards stepwise multiple linear regression analysis was used to develop a model for HbA1c prediction, setting the *p*-value for variable inclusion at *p* < 1.000. After adjusting for age, BMI, glucose tolerance, and metformin use, only adipsin remained significantly associated with HbA1c (p = 0.021). The final model included TNF-α, adipsin, age, BMI, and metformin status, while NGT was used as the reference group. The final model explained 86% of the variance in HbA1c (Table 5, Figure 1). To explore any multicollinearity and tolerance effects, the variance inflation factor (VIF) and tolerance (1/VIF) analyses were performed post-estimation (Table 6). None of the VIF values exceeded 10, therefore indicating little multicollinearity in the final model. In addition, since we found adipsin to be a significant predictor of HbA1c, we further analyzed the association (by Pearson correlation) of adipsin with other biomarkers. Adipsin showed a significant (*p* < 0.01) association with a few adipocytokines (Figure 2) but not with diabetes measures (body weight, BMI, glucose, and insulin).

## 4. Discussion

### 4.1. Adipokine Adipsin as a Predictor of HbA1c

The 14 measured adipocytokine biomarkers were chosen based on the literature review and data, including our previous data [39] showing involvement of these biomarkers in T2D development, progression, and complications [7,8,9,10,11,12,13,14,15,16,19,20,21]. The final model accounting for the most variance in chronic glycemic control as measured by HbA1c included adipsin and TNF-α. This is striking given that adipsin is one of the most abundant adipokines and a rate-limiting enzyme for the alternative complement system [51,52,53].

Specifically, we showed that adipsin was lower in T2D compared to NGT, which was similar to the majority of [40,54,55,56], but not all [10,19], previously published research. In addition, this study showed that 2 h post-glucose load adipsin was lower in T2D compared to NGT. These data were in agreement with our previous exploration of adipocytokine response to acute hyperglycemia [39]. We used randomly chosen samples from African-American men with prediabetes and vitamin D insufficiency who were participating in a vitamin D supplementation trial [44]. In the pilot ancillary analysis, the adipocytokines were compared between 60 vs. 0 min of OGTT. The findings relevant to the current study were that adipsin and complement-3 (C3), a component of the adipsin pathway [54], were lower at 60 vs. 0 min during OGTT. Specifically, in 20 men with an average age 57 years, BMI 31 kg/m^2^, and HbA1c 6.2%, adipsin was 4.26 ± 1.05 ng/mL at 60 min vs. 5.62 ± 1.44 ng/mL at 0 min (*p* < 0.001). In addition, in the group of 19 men from the same cohort with an average age 61 years, BMI 32 kg/m^2^, and HbA1c 6.1%, C3 was 0.35 ± 0.20 ng/mL at 60 min vs. 0.46 ± 0.22 ng/mL at 0 min (*p* < 0.038) [39]. These data from our current and previous explorations could be interpreted as suggesting inadequate response of the adipsin pathway to glucose load in T2D compared to normoglycemic participants. Although there had been no other previous studies evaluating circulating adipsin post-glucose load, our data could be interpreted as supporting previous translational studies suggesting that inadequate response of the adipsin pathway contributed to inadequate insulin secretion in a T2D animal model [54]. Correspondingly, a role of adipsin in insulin secretion was suggested by human data [40]. Specifically, adipsin positively correlated with the homeostasis model assessment of β-cell function (HOMA-β), the area under the curve (AUC) of the first phase insulin secretion, and acute insulin response in a group of Chinese individuals with various degrees of glucose tolerance, including newly diagnosed T2D [40]. Taken together, the data from previous and present research implicated failure of adipsin (or the adipsin pathway) to rise in response to acute hyperglycemia, which could be responsible at least in part for inadequate insulin increase in acute hyperglycemia in T2D.

The most unexpected finding of our study was that adipsin remained a significant predictor of HbA1c after multiple relevant adjustments in regression modeling. There were no previous studies that included multiple adipocytokines in modeling of HbA1c determinants and showing adipsin as a significant predictor of HbA1c. Previous studies measured only adipsin [56] or adipsin and IL-1β [40] and did not use HbA1c as a dependent variable in regression modeling. We preferred using HbA1c as a dependent variable in regression modeling because HbA1c represented the most clinically relevant biomarker of chronic glucose control. Contrary to our results, in regression modeling that used adipsin as a dependent variable, no association was found between adipsin and HbA1c in a cohort of 137 Chinese men and women with and without T2D [40]. The discrepancy between the current and previous results could be related to differences in populations and independent variables chosen for regression modeling. The current study, however, was in agreement with a previous investigation showing that circulating adipsin was negatively associated with insulin resistance, as calculated from a homeostasis model assessment of insulin resistance (HOMA-IR) [40,56], especially in participants with a BMI ≥ 25 kg/m^2^ or FPG ≥ 100 mg/dL [56]. 

The results of this study could be interpreted as supporting previous basic and translation research, providing mechanistic insight into the essential role of adipsin in T2D [54,57,58]. Adipsin, which was first identified as a component of the alternative complement cascade (called factor D) secreted by monocytes and macrophages [57,59], was later discovered as being produced by the white adipose cells [54] and by the gut epithelial Paneth cells [58]. Adipsin was shown to play a prominent role in regulation of inflammation and glucose metabolism [54,57,58]. Similar to the data in humans, in a mouse model adipsin was lower in T2D compared to non-diabetic mice [60], while in a mouse model of T2D development adipsin negatively correlated with glucose AUC during a glucose tolerance test [61]. Additionally, adipsin was shown to have a beneficial role in maintaining β-cell function [54]. Mice genetically lacking adipsin had glucose intolerance due to fasting and glucose-stimulated insulinopenia, while isolated islets from these mice had reduced glucose-stimulated insulin secretion [54]. Replenishment of adipsin to diabetic mice improved glucose control by increasing insulin secretion [54]. Treatment of diabetic mice with anti-diabetic agent (pioglitazone) resulted in increased gene expression for adipsin, PPAR-γ, and Glut-4 in adipose tissue, while multiple other biomarkers were not affected [62]. 

Further interrogation of mechanisms of adipsin action showed that adipsin activated the alternative complement cascade, generating C3a from C3, a small (approximately 10 KDa) cleavage fragment [54]. The C3a acted directly in isolated pancreatic islets as a potent insulin secretagogue, enhancing insulin secretion by 30–40% in the presence of high but not low glucose conditions. The C3a receptor was required for this beneficial effect. The action of C3a on islets was mediated by augmenting ATP levels, respiration, and cytosolic free Ca(2+) [54]. It was also shown that C3a could induce both proinflammatory and anti-inflammatory responses [63]. Furthermore, adipsin expression in the gut epithelial Paneth cells could be induced by *Lactobacillus* spp. (through TNF receptor-associated factor 2 (TRAF2) and TRAF6-mediated NF-kB signaling), and transplantation of these microbiota into germ-free mice resulted in increased adipsin in both the intestinal epithelium and serum [58]. Our data showing that adipsin was a significant predictor for HbA1c could be interpreted as being in agreement with these translational data showing an important role of adipsin in pathophysiology of diabetes.

Overall, the present study and previous human and translational research implicated the adipsin pathway in linking microbiota, inflammation, and adiposity to β-cell physiology and T2D pathogenesis. These data, if confirmed in other populations, could enable further understanding of the T2D pathophysiology and improve clinical decision-making based on specific risk profiles, as suggested by the data for cardiovascular disease [38]. 

### 4.2. Cytokine TNF-α as a Predictor of HbA1c

In addition to adipsin, TNF-α was associated with HbA1c. This result was in agreement with previous cross-sectional and prospective studies suggesting an association of TNF-α with T2D and risk for the incident T2D [7,8,9,12,41,64]. Our results, however, showed that the association between TNF-α and HbA1c was no longer significant (*p* = 0.088) after adjustment for age, BMI, and metformin use. Consistent with our observations, a cross-sectional study of the role of adipocytokines in T2D showed that TNF-α was higher in T2D (*n* = 63) compared to NGT (*n* = 304) in Mexican-Americans [41]. Nonetheless, in regression analysis, TNF-α was not a significant determinant of HbA1c after adjustment for age and BMI [41]. There were no other studies that investigated adipocytokines among other T2D covariates as possible determinants of HbA1c. Likewise, TNF-α was elevated in patients with incident T2D (*n* = 192 cases) compared to non-disease-developing controls (*n* = 384) in a nested case–control study within a prospective population-based European cohort (*n* = 27,548) [64]. In that study, however, TNF-α was not a significant predictor of the incident T2D after adjustment for BMI [64]. There was also a weak non-significant association between TNF-α and risk of T2D in the meta-analysis of 5 prospective studies comprising a total of 10,078 participants, including 2780 cases of incident T2D [7]. 

Of interest, two studies evaluated the effects of anti-TNF-α medications on glucose metabolism. A randomized double-blind trial showed no effect of a recombinant-engineered human TNF-α-neutralizing antibody (CDP571) compared to placebo on glucose homeostasis in obese T2D patients after 4 weeks [65]. A recent retrospective study analyzed the influence of TNF-α inhibitors (TNFi) and an anti-IL-6 receptor antibody tocilizumab (TCZ) on glucose metabolism in 221 patients with rheumatoid arthritis, including 109 patients with T2D [66]. The TNFi were infliximab, etanercept, and adalimumab. After 3 months of observation, HbA1c improved regardless of the presence of T2D. In a multivariate logistic regression analysis, use of TCZ but not TNFi remained a significant predictor of HbA1c reduction after adjustment for BMI, diabetes medications, and other relevant factors [66].

Mechanisms of TNF-α association with T2D likely at least partly involved direct stimulation by hyperglycemia TNF-α production and activity in adipose tissue [67,68,69]. This mechanistic insight was provided by human and animal studies. Hyperglycemic clamps in normal subjects, in whom endogenous insulin was suppressed by concomitant administration of somatostatin, induced an increase of circulating TNF-α and IL-6 [70]. In animal models and in obese humans, circulating TNF-α and TNF-α gene expression in adipose tissue were increased and associated with insulin resistance [67,68,69]. In summary, TNF-α, which is consistently considered a significant component of inflammation, appears to be one of multiple factors contributing to T2D, and its role in the complexities of T2D pathogenesis compels further elucidation.

The study had several strengths and limitations. The limitations were that the study involved a small group of homogenous participants that decreased the statistical power and generalizability to other populations, a cross-sectional design that precluded causal assessment, non-inclusion of some clinical characteristics (e.g., dietary assessment, sleep pattern), and non-inclusion of other adipocytokines with potential importance in T2D (e.g., leptin). The strengths of this study included the use of multiplex assays allowing exploration of multiple biomarkers, involvement of participants with variable glucose tolerance, and investigation of associations of adipocytokines with acute as well as chronic hyperglycemia.

## 5. Conclusions

This study showed for the first time that adipsin could be an independent predictor of HbA1c in African-American men with varied glucose tolerance and could play a significant role in T2D. After adjusting for age, BMI, glucose tolerance, and metformin use, only adipsin remained significantly associated with HbA1c (*p* = 0.021). Together with other relevant clinical characteristics and pathogenetic mediators, including age, BMI, use of metformin, and circulating adipsin and TNF-α, the model explained 86% of the HbA1c variability. Additional studies would be required to corroborate these observations and to provide mechanistic insights as well as enable adipsin-pathway-related discoveries of novel T2D treatment.

## Figures and Tables

**Table 1:** Participant characteristics by glucose status.

Variable	NGT (*n* = 20)	T2D (*n* = 21)		
		MF(+) (*n* = 16)	MF(−) (*n* = 5)	Combined
				(*n* = 21)
General				
Age, y	54.1 ± 5.8	60.1 ± 3.1 **	56.6 ± 6.7	59.2 ± 4.3 **
Body weight, kg	84.7 ± 11.5	105.5 ± 14.2 **	106.8 ± 14.7 **	105.8 ± 13.9 **
BMI, kg/m^2^	27.4 ± 3.3	35.4 ± 3.2 **	34.8 ± 2.4 **	35.3 ± 3.0 **
Charlson index	1.7 ± 1.1	3.4 ± 1.4 **	1.2 ± 0.6	2.9 ± 1.5 *
Number of all meds	8.3 ± 5.0	11.3 ± 4.9	2.2 ± 1.1	9.1 ± 5.0
Fasting Glycemic Indices				
HbA1c, %	5.3 ± 0.3	6.9 ± 0.5 **	6.8 ± 0.2 **	6.8 ± 0.4 **
Fasting glucose, mg/dL	93.6 ± 17.4	123.6 ± 27.8	119.8 ± 24.0	122.7 ± 26.4
Fasting Insulin, mIU/L	8.1 ± 3.9	35.9 ± 55.7	19.7 ± 5.1	32.1 ± 48.8
120 min Glycemic Indices				
Glucose 120 min, mg/dL	109.6 ± 42.0	198.1 ± 70.1	187.4 ± 100.0	195.6 ± 75.5
Insulin 120 min, mIU/L	43.3 ± 45.1	104.2 ± 63.7	67.9 ± 25.6	95.6 ± 58.5

Data are the mean ± standard deviation. Glucose and insulin were log-transformed prior to analysis. All group comparisons were adjusted for age and BMI where applicable. Note: *p*-values < 0.05 denote statistical significance, reference group is NGT. Fasting represents 0 min of OGTT. Abbreviations: meds, medications; NGT, normal glucose tolerance; T2D, type 2 diabetes; MF(+), taking metformin; MF(−), metformin-naïve; OGTT, oral glucose tolerance test; 120 min, 120 min post-glucose load during OGTT; * *p* < 0.05, ** *p* < 0.01.

**Table 2:** Cytokine levels at fasting and 120 min post-glucose load.

Variable	NGT (*n* = 20)	T2D (*n* = 21)		
		MF(+) (*n* = 16)	MF(−) (*n* = 5)	Combined
				(*n* = 21)
Fasting Cytokines				
GM-CSF	20.3 ± 15.3	21.4 ± 27.1	26.1 ± 28.9	22.5 ± 26.8
IL-10	7.3 ± 4.9	7.3 ± 6.7	6.4 ± 4.4	7.1 ± 6.1
IL-13	15.3 ± 41.1	9.7 ± 12.0 *	11.0 ± 14.3	10.0 ± 12.1 *
IL-6	6.3 ± 11.5	2.5 ± 2.8 **	3.7 ± 5.2 *	2.8 ± 3.4 **
IL-7	5.4 ± 3.2	6.9 ± 6.3	7.4 ± 6.9	7.0 ± 6.3
IL-8	74.9 ± 91.3	13.4 ± 21.8 **	9.4 ± 8.0 *	12.4 ± 19.3
TNF-α	14.5 ± 14.2	4.5 ± 2.7 **	5.0 ± 2.8 **	4.6 ± 2.7 **
120 min Cytokines				
GM-CSF	19.8 ± 14.8	20.8 ± 28.2	20.2 ± 21.6	20.6 ± 26.3
IL-10	7.2 ± 4.7	6.9 ± 6.9	6.4 ± 3.6	6.8 ± 6.3
IL-13	15.4 ± 41.5	9.8 ± 13.0 **	10.9 ±9.9	10.0 ± 12.3 **
IL-6	4.0 ± 9.3	2.7 ± 3.0 *	2.5 ± 3.4 *	2.6 ± 3.0 **
IL-7	5.1 ± 3.0	6.4 ± 6.9	5.7 ± 5.7	6.2 ± 6.5
IL-8	11.6 ± 14.9	5.0 ± 4.8 **	3.9 ± 1.3 *	4.7 ± 4.2 **
TNF-α	9.9 ± 10.3	4.3 ± 3.5 **	4.4 ± 2.2 **	4.3 ± 3.2 **

Data are the mean ± standard deviation. All values are expressed as pg/mL. All group comparisons were adjusted for age and BMI where applicable. Note: *p*-values < 0.05 denote statistical significance, reference group is NGT. Fasting represents 0 min of OGTT. Abbreviations: NGT, normal glucose tolerance; T2D, type 2 diabetes; MF(+), taking metformin; MF(−), metformin-naïve; OGTT, oral glucose tolerance test; 120 min, 120 min post-glucose load during OGTT; * *p* < 0.05, ** *p* < 0.01.

**Table 3:** Adipokine levels at fasting and 120 min post-glucose load.

Variable	NGT (*n* = 20)	T2D (*n* = 21)		
		MF(+) (*n* = 16)	MF(−) (*n* = 5)	Combined
				(*n* = 21)
Fasting Adipokines				
Adiponectin	60,785 ± 64,564	13,495 ± 15,055 *	12,227 ± 6467	13,193 ± 13,366 *
Lipocalin	897 ± 545	407 ± 204 *	399 ± 225 *	406 ± 203 *
Resistin	139 ± 89	89 ± 53	70 ± 56	84 ± 53
Adipsin	8758 ± 3037	7524 ± 4097 **	7469 ± 1512	7511 ± 3612 *
PAI-1 (total)	196 ± 101	168 ± 100 **	161 ± 53 *	166 ± 90 **
IP-10	409 ± 183	441 ± 243	286 ± 52	404 ± 222
MCP-1	434 ± 320	335 ± 147	438 ± 355	359 ± 20
120 min Adipokines				
Adiponectin	58,965 ± 67,994	10,190 ± 5774 **	9518 ± 1579 **	10,030 ± 5827 **
Lipocalin	568 ± 283	282 ± 144	273 ± 196	280 ± 152 *
Resistin	99 ± 60	66 ± 43	44 ± 42 *	61 ± 43 *
Adipsin	7947 ± 2773	6746 ± 5154 **	5364 ± 1579 **	6417 ± 4559 **
PAI-1 (total)	185 ± 111	141 ± 96 **	124 ± 84 **	137 ± 92 **
IP-10	345 ± 136	348 ± 207	245 ± 41.4	324 ± 125
MCP-1	384 ± 286	291 ± 131	396 ± 261	316 ± 170

Data are the mean ± standard deviation. All values are expressed as pg/mL. Adipocytokines were log-transformed prior to analysis. All group comparisons were adjusted for age and BMI where applicable. Note: *p*-values < 0.05 denote statistical significance, reference group is NGT; * *p* < 0.05, ** *p* < 0.01. Abbreviations: NGT, normal glucose tolerance; T2D, type 2 diabetes; MF(+), taking metformin; MF(−), metformin-naïve; OGTT, oral glucose tolerance test; 120 min, 120 min post-glucose load during OGTT.

**Table 4:** Independent adipocytokine associations with HbA1c in the fasted state (*n* = 41).

Independent Variable	β-Coefficient (SE)	*p*-Value
GM-CSF	−0.14 (0.08)	NS
IL-10	−0.02 (0.07)	NS
IL-13	−0.05 (0.07)	NS
IL-6	−0.08 (0.07)	NS
IL-7	−0.01 (0.11)	NS
IL-8	−0.14 (0.06)	0.032
TNF-α	−0.42 (0.09)	<0.001
Adiponectin	-0.28 (0.09)	0.003
Lipocalin	−0.42 (0.14)	0.005
Resistin	−0.33 (0.10)	0.003
Adipsin	−0.0001 (0.00002)	<0.001
PAI-1 (total)	−0.67 (0.14)	<0.001
IP-10	0.11 (0.21)	NS
MCP-1	−0.02 (0.18)	NS

Data are β-coefficient values (standard error, SE). All variables were log-transformed prior to analysis. Each line represents a regression, with HbA1c as the dependent variable and an adipocytokine as an independent variable, adjusted for BMI and age.

**Table 5:** Backwards stepwise multiple linear regression for HbA1c in the fasted state (*n* = 41).

Independent Variables	Beta (SE)	95% CI	*p*-Value
TNF-α	−0.16 (0.09)	−0.34, 0.03	0.088
Adipsin	−0.43 (0.14)	−0.72, −0.15	0.004
Age (years)	0.03 (0.01)	0.004, 0.05	0.025
BMI (kg/m^2^)	0.04 (0.02)	0.002, 0.08	0.036
NGT	Reference	-	-
MF(−)	0.93 (0.26)	0.40, 1.45	0.001
MF(+)	0.86 (0.26)	0.32, 1.10	0.003

Data are β-coefficient values (standard error, SE) and 95% confidence intervals (CI). Adipsin and TNF-α were log-transformed. Variables with *p* < 1.000 from Table 3 were used. Variables were removed if *p* > 1.000. The model predicted 86% variability of HbA1c (R^2^-adjusted 0.86) for the whole group. Abbreviations: NGT, normal glucose tolerance; MF(−), T2D metformin-naïve, MF(+), T2D using metformin.

**Table 6:** Variance inflation factors (VIF) for multiple linear regression.

Variable	VIF	1/VIF
TNF-α	1.93	0.519311
Adipsin	1.22	0.818808
Age (years)	1.49	0.670785
BMI (kg/m^2^)	3.56	0.280930
NGT	-	-
MF(−)	2.72	0.367760
MF(+)	6.11	0.163683

VIF < 10.0 indicates limited multicollinearity, while 1/VIF < 1.0 indicates limited collinearity. Adipsin and TNF-α were log-transformed.
